# Computationally Efficient Method for Determining Limiting Velocities of Edge Dislocations in Anisotropic Crystals

**DOI:** 10.3390/ma19153180

**Published:** 2026-07-25

**Authors:** Daniel N. Blaschke

**Affiliations:** Los Alamos National Laboratory, Los Alamos, NM 87545, USA; dblaschke@lanl.gov

**Keywords:** dislocations in crystals, dislocation mobility, crystal plasticity

## Abstract

The continuum limit theory of dislocations in crystals predicts divergences in the elastic energy at crystal geometry-dependent limiting velocities $v_{L}$. The $v_{L}$ separate subsonic, transonic, and supersonic dislocation glide regimes, and are therefore important for material strength models at high strain rates. Although it is known how to calculate these limiting velocities, there is one special case—edge dislocations with reflection symmetry, but non-vanishing elastic constants $c_{16}^{\prime }$ or $c_{26}^{\prime }$—where previous methods have been notoriously slow. In this paper, we address this deficiency by deriving a computationally efficient method for determining the $v_{L}$ of edge dislocations with reflection symmetry that is two orders of magnitude faster than the previous method.

## 1. Introduction and Background

The flow stress, and thereby the deformation rate, of crystalline materials such as metals is strongly influenced by the glide speed of dislocations [1,2,3,4,5,6,7,8,9,10,11,12]. If dislocations reach a limiting velocity, more dislocations need to be generated in order to accommodate higher deformation rates. Even if transonic or supersonic dislocation glide is possible, the limiting velocities constitute a hard-to-overcome barrier. The limiting velocities $v_{L}$ can be determined from the continuum-limit theory of dislocations, which predicts divergences in the elastic energy at crystal geometry-dependent $v_{L}$; see the review article by [13]. Dislocation-core regularization [14,15] and lattice treatments [16] have shown that these limiting velocities can be overcome in principle, and molecular dynamics simulations have predicted the existence of supersonic (single) dislocations [17,18,19,20,21,22].

Recent measurements of diamond have also shown that supersonic dislocations exist [23]. With the recent advances in femtosecond x-ray radiography [24,25], this might be just the first of several future discoveries related to transonic dislocations in crystals. Aside from the questions around how dislocations can overcome (even soft) gliding velocity barriers, it is essential to know the values of these $v_{L}$, as the flow stress will increase non-linearly close to them [26]. In a larger crystal plasticity finite element (CPFE) [4,5,10,27] or discrete dislocation dynamics (DDD) simulation [7,8,11,28], limiting velocities will need to be calculated fast and on-the-fly, as they depend on the material density and elastic constants, and are thereby temperature- and pressure-dependent.

Based on the so-called integral method [29,30] of computing the dislocation displacement field, Barnett et al. [31] presented a computationally efficient method of how to determine dislocation-limiting velocities in general anisotropic crystals for arbitrary slip systems and dislocation character angles. In general, there are three (slip system and dislocation character-dependent) gliding velocities where the dislocation self-energy diverges. These limiting velocities thus separate dislocation glide into a ‘sub-sonic’, two ‘transonic’, and a ‘supersonic’ regime, where the lowest of these $v_{L}$ separates the ‘sub-sonic’ from the first ‘transonic’ regime of dislocation glide. However, Barnett’s method does not account for special cases where the plane perpendicular to the dislocation line is a reflection plane. In this case, the differential equations for edge and screw dislocations decouple. Therefore, the screw dislocation has only one limiting velocity and the edge dislocation has two limiting velocities. Analytic solutions exist for the $v_{L}$ of screw dislocation in this case, as well as for the edge dislocation, if elastic constants $c_{16}=0=c_{26}$ in the rotated coordinate system where the $\hat{z}$ axis is aligned with the dislocation and the $\hat{y}$ axis is aligned with the slip plane are normal; see [32,33,34,35,36] as well as the review article by [13].

The one case that is not well covered is that of edge dislocation with reflection symmetry and non-vanishing $c_{16}$ and/or $c_{26}$. Python code that can be used to calculate the limiting velocity in this case was previously implemented in the present author’s code PyDislocDyn 1.3.5 [37,38], and the method was based on Teutonico’s 1961 paper [33] using a combination of symbolic (sympy) and numeric methods.

In this paper, we derive a specialized version of Barnett’s 1973 algorithm [31] adapted to the decoupled two-dimensional case of edge dislocation with reflection symmetry. This improved method is roughly two orders of magnitude faster to compute than the previous one, which was based on Teutonico’s work [33].

We start by reviewing the general three-dimensional Barnett algorithm, which we subsequently simplify for the two-dimensional case of edge dislocation with reflection symmetry. It must also be noted that, in the case of reflection symmetry, there are subtle cancellations in the numerator of the dislocation field that depend on polar angle ϕ [13,36]. Therefore, the true limiting velocity can be higher than min$\left(v_{\mathrm{limit}}(\phi )\right)$ computed from the full three-dimensional determinant. In other words, the Barnett method fails in these special cases, which showcases the necessity of a two-dimensional version specialized to the case of edge dislocation with reflection symmetry. Despite the simplicity of the resulting equations, this new method has not appeared in the literature, to the author’s best knowledge.

## 2. Review of Barnett’s Method

The dislocation displacement field $u_{i}$ follows from the differential equations

(1) $$\begin{array}{cccc} \partial_{i}\sigma_{ij} & =\rho {\ddot{u}}_{j}\;, & \;\sigma_{ij} & =C_{ijkl}\partial_{l}u_{k}\;, \end{array}$$

where $C_{ijkl}$ is the tensor of second order elastic constants (SOECs). The established numerically efficient method of computing $u_{i}$ in the steady state limit, where the dislocation depends on time only via the linear combination x−vt, is the so-called integral method [29,30]:

(2) $$\begin{array}{cc} u_{j}\left(r,\phi \right) & =-\frac{b_{l}}{2\pi }\left\{S_{jl}\ln r-S_{il}\int_{0}^{\phi }{\left[{\left(nn\right)}^{-1}\left(nm\right)\right]}_{ji}d\phi^{\prime }-B_{il}\int_{0}^{\phi }{\left(nn\right)}_{ji}^{-1}d\phi^{\prime }\right\}\;,\\ S & =-\frac{1}{2\pi }\int_{0}^{2\pi }{\left(nn\right)}^{-1}\left(nm\right)\;d\phi \;,\\ B & =-\frac{1}{2\pi }\int_{0}^{2\pi }\left[\left(mn\right){\left(nn\right)}^{-1}\left(nm\right)-\left(mm\right)\right]d\phi \;, \end{array}$$

with the short-hand notation $\left(ab\right):=\left(a_{i}C_{ijkl}b_{j}-\rho v_{i}a_{i}\delta_{jk}v_{l}b_{l}\right)$, and we use the Einstein summation convention, i.e., indices appearing twice are summed over. We note that all expressions in (2) are π-periodic in ϕ. The vectors $m_{i}$ and $n_{i}$ are orthonormal to one another and lie in the plane perpendicular to the dislocation line; for details, we refer to the excellent review article by Bacon, Barnett, and Scattergood [29].

Since Equation (2) above depends on the inversion of matrix (nn), a general mixed-character dislocation exhibits a divergence in its elastic self-energy whenever the following determinant is zero:

(3) $$\begin{array}{c} \det\left[n_{i}C_{ijkl}n_{l}-\rho {\left(v\cos\phi \right)}^{2}\delta_{jk}\right]=0\;, \end{array}$$

where ϕ is the angle between velocity vector $\vec{v}$ and $\vec{n}$. The latter vector lies in the plane perpendicular to the dislocation line and ϕ is the polar angle in that plane. The determinant above can be solved in terms of v(ϕ) and has three ϕ-dependent solutions in general, which are the roots of a cubic polynomial [31]:

(4) $$\begin{array}{cc} \; & x^{3}+bx^{2}+cx+d=0\;,\\ \; & b=-\mathrm{tr}(\vec{n}\cdot C\cdot \vec{n})=-\sum_{j=1}^{3}n_{i}C_{ijjl}n_{l}\;,\\ \; & c=\frac{1}{2}\left[b^{2}-\mathrm{tr}\left({\left(\vec{n}\cdot C\cdot \vec{n}\right)}^{2}\right)\right]=\frac{1}{2}\left[b^{2}-n_{i}C_{ijjl}n_{l}n_{k}C_{kjjr}n_{r}\right]\;,\\ \; & d=-\det(\vec{n}\cdot C\cdot \vec{n})\;. \end{array}$$

The transformation x=y−b/3 eliminates the quadratic term leading to the simpler equation

(5) $$\begin{array}{cc} \;y^{3}+3py+2q=0\;, & \\ p=\frac{1}{3}\left(c-b^{2}/3\right)\;, & \;q=\frac{d}{2}-\frac{bc}{6}+\frac{b^{3}}{27}\;. \end{array}$$

The solutions, which are real for the elasticity problem at hand (and can be written in different ways), are

(6) $$\begin{array}{ccc} y & =2\sqrt{-p}\cos\left(\frac{\psi +2k\pi }{3}\right) & \;k=0,1,2\;,\\ \cos\psi & =\frac{-q}{\sqrt{-p^{3}}}\;, & \\ \rho {\left(v\cos\phi \right)}^{2} & =x=y-b/3\;. & \end{array}$$

Thus, the three limiting velocities of a dislocation of mixed character angle are determined by minimizing the three branches v(ϕ) over the interval [0,π] (since $\vec{n}$ is a function of ϕ and several terms in π-periodic Equation (2) are integrated over ϕ).

## 3. Dislocations with Reflection Symmetry

If the plane perpendicular to the dislocation line is a reflection plane, the differential equations for the screw and edge dislocations decouple. Hence, one is left with a single differential equation for pure screw dislocations and two coupled differential equations for pure edge dislocations. The equation for the screw dislocation can be solved analytically for the single limiting velocity [13,33,36]. An analytic solution for the two limiting velocities of the edge dislocation only exists for a special case where one additional condition is met [13,33]: After rotating into a coordinate system where the $\hat{z}$ axis is aligned with the dislocation and the $\hat{y}$ axis is aligned with the slip plane normal, the elastic constants $c_{16}=0=c_{26}$ must vanish.

The more general case, where $c_{16}$ and $c_{26}$ are non-zero, must be solved numerically. A method following Teutonico’s 1961 paper [33] was put forward in Ref. [13] and was implemented using the Python code PyDislocDyn [37,38]. However, because that implementation uses a combination of symbolic (sympy) and (scipy) minimization routines, it is fairly slow and cannot be readily ported to C or Fortran.

On the other hand, the three-dimensional general method of Barnett, reviewed above in Section 2, fails in this high-symmetry case. The reason for this is that the terms in the numerator of the dislocation field cancel out the divergence from the vanishing determinant for certain polar angles ϕ. In other words, it becomes unclear regarding what (limited) range of polar angles ϕ one must minimize in this case. A prime example is the {112} slip planes in body-centered cubic (bcc) crystals, where reducing the minimization-interval to [π/2,π] leads to the correct lowest limiting velocity for edge dislocations—at least for the handful of cases checked by the author.

A purely numerical solution along the lines of Section 2, but reduced to the two-dimensional problem at hand, is therefore in order. We stress that the new method we are about to derive does not require the tuning of any interval; all functions are π-periodic, and, therefore, minimization will be strictly over ϕ∈[0,π] below.

The first step is to rotate all quantities into a coordinate system aligned with the dislocation. The rotation matrix we need for this purpose is determined by stacking the following three row vectors into a matrix, i.e.,

(7) $$\begin{array}{cc} U & =\left(\begin{array}{c} {\hat{m}}_{0}^{T}\\ {\hat{n}}_{0}^{T}\\ {\hat{t}}^{T} \end{array}\right)\;, \end{array}$$

where ${\hat{m}}_{0}$ is the slip direction, ${\hat{n}}_{0}$ is the slip plane normal, and $\hat{t}$ the edge dislocation line direction. Hence, $U\cdot {\hat{m}}_{0}=\hat{x}$, $U\cdot {\hat{n}}_{0}=\hat{y}$, and $U\cdot \hat{t}=\hat{z}$. The tensor of the second-order elastic constants, which is always measured in crystal coordinates, is rotated into coordinates aligned with the dislocation via

(8) $$\begin{array}{c} C_{ijkl}^{\prime }=U_{im}U_{jn}U_{ko}U_{lp}C_{mnop}\;. \end{array}$$

**Figure 1:**
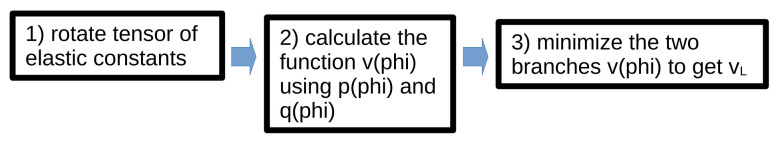
Flowchart summarizing the three main steps necessary to calculate the limiting velocity of an edge dislocation with reflection symmetry using the new method presented here. Step 1 is captured by Equation (8) and step 2 two by Equation (14) (with *p*, and *q* given in (13)), which is then minimized in step 3.

The edge dislocation displacement field $u_{i}=U_{ij}{\tilde{u}}_{j}$ in this coordinate system has only two non-vanishing components, $u_{1}$ and $u_{2}$ (i.e., displacements only in the *x* and *y* directions). Furthermore,

(9) $$\begin{array}{c} {\vec{n}}^{\prime }=U_{ij}n_{j}=\left(\begin{array}{c} \cos\phi \\ \sin\phi \\ 0 \end{array}\right) \end{array}$$

because of (7). The determinant of the two-dimensional matrix we must consider for the pure edge dislocation is hence

(10) $$\begin{array}{c} \det\left[n_{i}^{\prime }C_{i\alpha \beta j}^{\prime }n_{j}^{\prime }-\rho {\left(v\cos\phi \right)}^{2}\delta_{\alpha \beta }\right]=0\;, \end{array}$$

where i,j∈[1,2,3] and free indices α and β∈[1,2] reflect the two-dimensional nature of the matrix, whose determinant we presently consider. The resulting quadratic polynomial equation in $X=\rho {\left(v\cos\phi \right)}^{2}$ is

(11) $$X^{2}-2pX+q=0\;,$$

(12) $$p=\frac{1}{2}\left(n_{i}^{\prime }C_{i11j}^{\prime }n_{j}^{\prime }+n_{i}^{\prime }C_{i22j}^{\prime }n_{j}^{\prime }\right)\;,$$

(13) $$q=n_{i}^{\prime }C_{i11j}^{\prime }n_{j}^{\prime }n_{k}^{\prime }C_{k22l}^{\prime }n_{l}^{\prime }-n_{i}^{\prime }C_{i12j}^{\prime }n_{j}^{\prime }n_{k}^{\prime }C_{k21l}^{\prime }n_{l}^{\prime }\;,$$

where, for the sake of clarity, the sums over the 2D Greek indices have been written out explicitly. The solutions are $X=p\pm \sqrt{p^{2}-q}$ (which are both real for the elasticity problem at hand). Hence, the two limiting velocities for pure edge dislocations are

(14) $${\left(v_{L}\right)}^{2}=\min\left[\frac{p}{\rho \cos^{2}\phi }\pm \sqrt{p^{2}-q}\right]\;,$$

which must be numerically minimized over ϕ∈[0,π] (since all expressions involved are π-periodic). The lower of the two limiting velocities separates the subsonic from the transonic regime, whereas the higher limiting velocity separates the transonic from the supersonic regime; see [13]. The main steps required to implement this method numerically are summarized in the flowchart in Figure 1.

**Figure 2:**
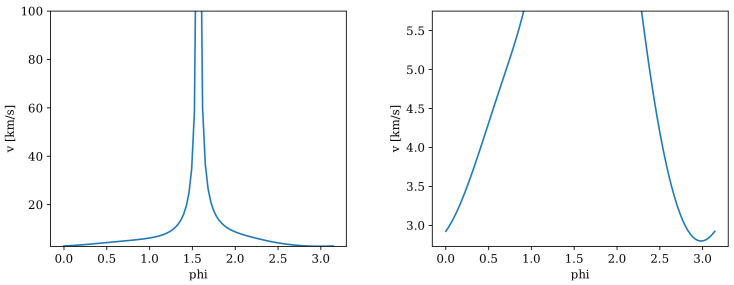
The square root of the smaller branch of (14) prior to minimization for the example of a {112} slip plane for bcc iron. At ϕ=π/2 (where cosϕ=0), the function diverges, as seen in the left panel. In the right panel, we show the same function again, but zoomed in with respect to the *y*-axis in order to see more clearly where the function’s minimum lies. In this example, the lowest $v_{L}=2.7999$ km/s for the edge dislocation and this minimum is found at angle ϕ≈0.949495π.

Figure 2 shows an example of what the function we need to minimize may look like, i.e., it shows a plot of the square root of the smaller branch of (14) prior to minimization as a function of ϕ using the example of bcc iron for the {112}-slip plane. The material density and elastic constants for Fe (and a number of other metals) are listed in Table 1.

## 4. Results—Validating the New Method

The new method has been implemented in PyDislocdyn 1.3.5 [37,38] and has been verified to yield comparable results to the slower previous method in Refs. [13,33]. As an additional consistency check, one can calculate the line tension [40] and/or drag coefficient from phonon wind [41,42] close to the limiting velocity to see that they quickly increase in value as the correct limiting velocity is approached. Furthermore, the new method works equally well for the special case of $c_{16}=0=c_{26}$ or even the isotropic limit (where analytic expressions exist). In fact, PyDislocDyn uses this numerical method if $c_{16}=0=c_{26}$ and the user requested both limiting velocities (rather than just the lowest one) for an edge dislocation with reflection symmetry.

We proceed to examine a few examples with elastic constants and material densities listed in Table 1. Review article [13] tells us that the {112} slip planes in bcc crystals fulfill the reflection symmetry requirement with non-vanishing (rotated) elastic constants $c_{16}$, $c_{26}$. Table 2 compares the results for the lowest limiting velocity of edge dislocations in a selection of bcc crystals using both the new (fast) method presented here, as well as the much slower one based on Teutonico’s paper.

Tetragonal crystals also exhibit edge dislocations with reflection symmetry in many of their slip systems, most of which have vanishing elastic constants $c_{16}$, $c_{26}$. In the case of β-tin, we identified one slip system in the list presented in Ref. [43], which features a reflection symmetry for its edge dislocation with non-vanishing $c_{16}$ and $c_{26}$. This is the one we included in the last row of our examples in Table 2. In all cases, the results of both methods are comparable, although very small differences are expected, as both algorithms rely on numeric minimization. PyDislocDyn uses scipy’s default options when employing the minimization function scipy.optimize.direct(), which is an implementation of the DIviding RECTangles algorithm in Ref. [44].

For all of the metals in Table 2, the run-time of the Python implementation of PyDislocDyn on an M3 CPU was measured to be between 120–150 ms for the ‘slow’ method and 0.5–0.6 ms for the ‘fast’ method. Furthermore, the latter can be implemented in a compiled language such as Fortran or C, whereas the ‘slow’ method requires symbolic manipulations with sympy.

Having established that the ‘fast’ and ‘slow’ methods give comparable results (see Table 2), it remains to show an example where the 3D Barnett method in Section 2 fails. Again choosing iron as our example (using the values listed in Table 1), the new method shows that the two limiting velocities of the edge dislocation in the {112} slip plane are 2799.87 m/s and 6409.70 m/s. The 3D Barnett method in Section 2, on the other hand, yields three values, namely 2629.48 m/s, 2925.01 m/s, and 6409.70 m/s (i.e., only the highest one is correct).

Computing the drag coefficient from phonon wind using PyDislocDyn at three different velocities (one low and the other two slightly below the predicted lowest limiting velocities of the Barnett and the ‘new’ methods), we find, for the drag coefficient *B* of an edge dislocation in a {112} slip plane of iron,

$$\begin{array}{cc} B(v=300\;m/s) & =0.0215\;\mathrm{mPa}\;s,\\ B(v=2629.48\;m/s) & =0.1221\;\mathrm{mPa}\;s,\\ B(v=2799.8\;m/s) & =144.99\;\mathrm{mPa}\;s\;, \end{array}$$

confirming that our new method (and not Barnett’s) yields the correct result in this case, since the drag coefficient only diverges at the correct limiting velocity [42], which is 2799.87 m/s in this example.

## 5. Conclusions

We have derived a computationally efficient fast method of determining the limiting velocities of gliding edge dislocations with reflection symmetry, but non-vanishing elastic constants $c_{16}^{\prime }$ or $c_{26}^{\prime }$—the one special case where an efficient method was previously lacking in the literature.

A reference implementation of how to compute limiting velocities of gliding dislocations for all cases is given within the open source code PyDislocDyn [37,38] developed by the present author. The method presented here for pure edge dislocations in anisotropic crystals, where the plane perpendicular to the dislocation is a reflection plane, constitutes the last “missing piece” needed to calculate all dislocation limiting velocities for all slip systems and dislocation characters numerically efficient and without the need for any symbolic (sympy) routines. It is therefore now possible to implement on-the-fly calculations of dislocation-limiting velocities into larger simulations codes, such as crystal plasticity finite element (CPFE) or discrete dislocation dynamics (DDD) simulations. An implementation in Fortran (in addition to Python) has therefore been included in the current development version of PyDislocDyn, i.e., a Fortran library (or frontend) can be compiled and linked to (called from) other codes where speed is essential.

## Figures and Tables

**Table 1:** Material densities and elastic constants of a selection of bcc metals and body-centered tetragonal (bct) β-tin are shown. The values are taken from Ref. [39], see also [37,38] and the references therein. The last row shows the Zener anisotropy ratio $A_{Z}=2c_{44}/\left(c_{11}-c_{12}\right)$ for the cubic metals.

	Cr	Fe	K	Mo	Nb	Ta	V	W	Sn
rho [g/ccm]	7.15	7.87	0.89	10.20	8.57	16.40	6.00	19.30	7.287
c11 [GPa]	339.80	226.00	3.70	463.70	246.50	260.20	228.70	522.39	75.29
c12 [GPa]	58.60	140.00	3.14	157.80	134.50	154.40	119.00	204.37	61.56
c44 [GPa]	99.00	116.00	1.88	109.20	28.73	82.55	43.20	160.58	21.93
c13 [GPa]	-	-	-	-	-	-	-	-	44.00
c33 [GPa]	-	-	-	-	-	-	-	-	95.52
c66 [GPa]	-	-	-	-	-	-	-	-	23.36
$A_{Z}$	0.70	2.70	6.71	0.71	0.51	1.56	0.79	1.01	-

**Table 2:** We compare the limiting velocities in units of m/s calculated with both methods (i.e., the slow one based on Teutonico, and the fast one presented here). The last column shows the difference between the two methods, normalized by the ‘slow’ one. Small differences are expected, as both algorithms rely on numeric minimization.

	Slip System	$v_{\lim}^{\mathrm{edge}}$ [m/s], Fast	$v_{\lim}^{\mathrm{edge}}$ [m/s], Slow	$\left(\frac{\mathrm{fast}}{\mathrm{slow}}-1\right)$
Cr, (bcc)	{112} $<11\bar{1}>/2$	4092.802073	4092.802073	$4\;\times \;10^{-15}$
Fe, (bcc)	{112} $<11\bar{1}>/2$	2799.874438	2799.874438	$4\;\times \;10^{-12}$
K, (bcc)	{112} $<11\bar{1}>/2$	864.896309	864.896309	$8\;\times \;10^{-12}$
Mo, (bcc)	{112} $<11\bar{1}>/2$	3591.693933	3591.693933	$4\;\times \;10^{-15}$
Nb, (bcc)	{112} $<11\bar{1}>/2$	2102.524225	2102.524225	$4\;\times \;10^{-14}$
Ta, (bcc)	{112} $<11\bar{1}>/2$	1932.919026	1932.919026	$1\;\times \;10^{-12}$
V, (bcc)	{112} $<11\bar{1}>/2$	2883.439916	2883.445660	$-2\;\times \;10^{-6}$
W, (bcc)	{112} $<11\bar{1}>/2$	2875.030768	2875.030767	$3\;\times \;10^{-11}$
Sn, (bct)	{011} $<01\bar{1}>$	1681.278992	1681.278992	$1\;\times \;10^{-13}$

## Data Availability

The original contributions presented in this study are included in the article. Further inquiries can be directed to the corresponding author.
